# Research on reliability index and failure probability of inherent defect insurance from the insurance perspective

**DOI:** 10.1016/j.heliyon.2024.e26160

**Published:** 2024-02-14

**Authors:** Zeyu Chen, Xikang Yan, Lida Wang, Qinyu Luo, Yunhan Yan, Tian Qiu, Peng Cheng

**Affiliations:** a School of Civil Engineering and Transportation, Hebei University of Technology, Tianjin 300401, China; b Hebei Civil Engineering Technology Research Center, Hebei University of Technology, Tianjin 300401, China; c Hebei Sustainable Rural Construction Research Center, Hebei University of Technology, Tianjin 300130, China; d School of Management, Tianjin University of Commerce, Tianjin 300134, China; e School of Architecture and Art Design, Hebei University of Technology, Tianjin 300130, China

**Keywords:** Inherent defect insurance, PSO-LSSVR model, Performance function, Failure probability, Insurance risk

## Abstract

With the continuous improvement of people ‘s living standards, people have put forward higher requirements for the safety and comfort of housing. Therefore, Inherent Defect Insurance, a financial method to guarantee the quality of construction projects, has also emerged. At present, China ‘s Inherent Defect Insurance has been gradually promoted, but its claim mechanism has not been analyzed and studied. From the perspective of construction engineering, this paper first makes a bibliometric analysis of the influencing factors of insurance claims that may be caused by construction engineering quality through VOSViewers, and the evaluation index system of inherent defects is constructed. Then, according to the influencing factors, the PSO-LSSVR model is adopted to fit the performance function of the inherent defects. Finally, based on the reliability design principle of engineering structure, the reliability index and failure probability of Inherent Defect Insurance are derived from the performance function of inherent defects. This paper also analyzes its application in insurance practice and determines the relationship between the number of insurance underwriting policies and the initial reserve of insurance at a certain risk level. This paper studies the probability of Inherent Defect Insurance by constructing the reliability model of inherent defect risks in construction quality, and analyzes the anti-risk ability of insurance companies from the perspective of claim, which provides scientific analysis methods and theoretical basis for the scientific decision-making of insurance companies.

## 1 Introduction

With the continuous development of the construction industry, the problem of inherent defects in construction projects has gradually become an important aspect of quality of life. The improvement in living standards also puts forward higher requirements for housing quality. According to the statistics of an insurance company in China, the quality problems of construction projects in the insurance projects undertaken by the company, including the foundation, main structure, thermal insulation, waterproofing, electrical and decoration, are frequentand seriously affects the quality of life of residents and the rights and interests of consumers [1].

Inherent Defect Insurance (hereinafter referred to IDI) refers to the insurance that the construction enterprise handles according to the insurance scope and period stipulated in the contract, and the insurance company bears liability for the loss caused by inherent defects in the project quality [2]. The IDI originated in France, prevailed in Europe, and gradually developed in the Americas, Asia and Africa. It is an indispensable insurance service in the construction industry and has a history of more than 30 years. Currently, IDI has a mature guarantee mechanism that is applicable worldwide [3].

**Fig. 1:**
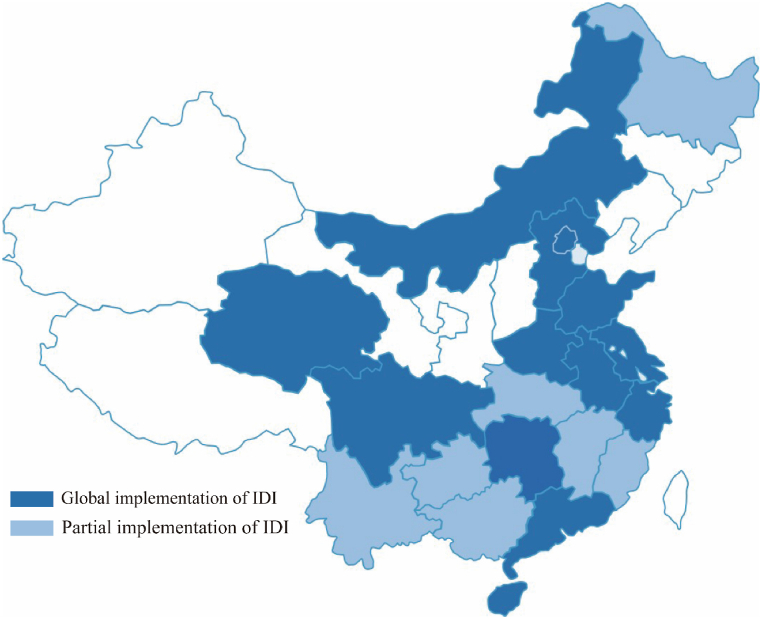
IDI implementation diagram of provinces and cities in China.

IDI has been piloted in major cities in China now. According to the information disclosed by Shanghai, by the end of May 2023, there were 1695 residential projects that purchased IDI insurance in the Shanghai, with an insured amount of CNY 696.4 billion and an insured area of 176 million square meters during the two-year pilot period [4]. Similarly, according to the data disclosed by Guangzhou in June 2023, as a pilot promoted in some areas, the number of IDI in Guangzhou has reached 137, with a total premium of 985.53 million [5]. Currently, many provinces and cities in China have introduced policies related to IDI; the distribution of these provinces and cities is shown in Fig. 1. In Fig. 1, the dark blue part represents the provinces that implement IDI in the entire region, and the light blue part represents the provinces that implement IDI in some regions.

Many scholars have analyzed the factors influencing the qualityof construction engineering. Vahedifard constructed an assessment model under various conditions and scenarios to analyze and assess the impact of extreme climate conditions on infrastructure in the future [6]. In this research, the risks to infrastructure in different historical and future climate scenarios were assessed and compared using Monte Carlo sampling methods for scenario simulations [6]. Carretero-Ayuso analyzed the claims arising from quality problems in 496 buildings in Spain and determined the most likely influencing factors in each facility through statistical analysis. This indicates that climatic factors are among the main influencing factors [7]. Based on the actual situation of prefabricated buildings in six climate zones in Australia, Naji analyzed the impact of climate on the quality of prefabricated buildings [8]. In addition, Amoah believes that the degree of national development will affect the supervision and management levels of construction projects and, accordingly, that there will be different assessment standards, which will have an impact on the quality of construction projects [9]. By comparing the quality of construction projects in Bosnia, Herzegovina and Croatia, which are also neighbours of the former Yugoslavia, and taking the two countries as representatives of developing and developed countries, respectively, Ljevo concluded that the degree of regional development has a significant impact on the quality of local projects [10]. Fan used data mining algorithms to analyze the defects in 1015 inspection items and the correlation between the quality level and factors such as policy environment and government service level [11]. In addition, a classification model for quality prediction was developed using an intelligent algorithm, which provides a reference for effective engineering quality management [11].

Although many scholars have described the possible causes of construction engineering problems from various angles, quantitative actuarial models are still lacking. It causes insurance companies to focus on the risks of insurance projects and lack the motivation to implement IDI, which makes the lower development level of IDI and has not been fully promoted nationwide since the first IDI policy was introduced in Shanghai in 2014. In real life, engineering quality problems do not occur randomly, but are related to many influencing factors. Therefore, a better analysis of the impact of various related factors on inherent defects have become an urgent problem to be solved.

Currently, the relevant scholars ' research on the impact on the quality of construction projects is mostly in the study of the overall impact of construction projects. However, different sub-projects involve different influencing factors, and the study of the overall impact of construction projects is difficult to conduct in-depth analysis of the mechanism. Ma put forward the idea of aggregating each sub-defect model into the engineering quality defect model of the whole construction project through BIM model [12]. However, this research also has not comprehensively summarized all the factors that affect the quality of construction projects. The influencing factors of the construction project can be carried out from two aspects: the climatic conditions and the policy conditions.

For the climatic conditions, many scholars have conducted relevant research on the factors that cause quality defects in construction projects. Climatic conditions are the most direct factors affecting the quality of construction projects, and temperature should be considered first [[13], [14], [15]]. Extreme temperatures may reduce the durability of concrete, leading to engineering quality problems [16,17]. Similarly, Qi Yuting also mentioned this point in the study of energy-saving renovation of existing urban residential buildings, and summarized the potential defects of engineering quality [18,19]. In addition to temperature, humidity is also one of the natural factors affecting the quality of construction projects. The study of coastal construction projects shows this point [[20], [21], [22]]. The coastal climate will have an impact on the forming of building foundation materials, which will affect the construction project [23,24].

In addition to the climatic conditions such as temperature and humidity, the policy conditions are also a major factor affecting the inherent defects. The impact of the policy environment on the quality of the project is particularly important, and the construction industry ‘s total quality management (TQM) principles and national building codes have played a significant role [25,26]. Similarly, the information data of public construction projects in Ethiopia also analyzed such a conclusion [27,28]. In addition, controlling the conduction of quality behavior risk is the key to effectively supervise and ensure the quality of construction projects [[29], [30], [31]]. This is also confirmed by the study of different construction modes of prefabricated buildings [32].

Although various factors have been shown to have an impact on construction projects Through empirical analysis, the intrinsic link between construction problems and these influencing factors has not yet been excavated. This paper aims to construct a quantitative actuarial model and further determine its distribution on the basis of the factors affecting the quality of construction projects. The failure probability of IDI by constructing a mathematical model can provide a tool for the scientific decision-making of insurance companies and the promotion of IDI, which will effectively enhance the management of construction projects and improve the quality of construction projects to improve the living standards of residents.

**Fig. 2:**
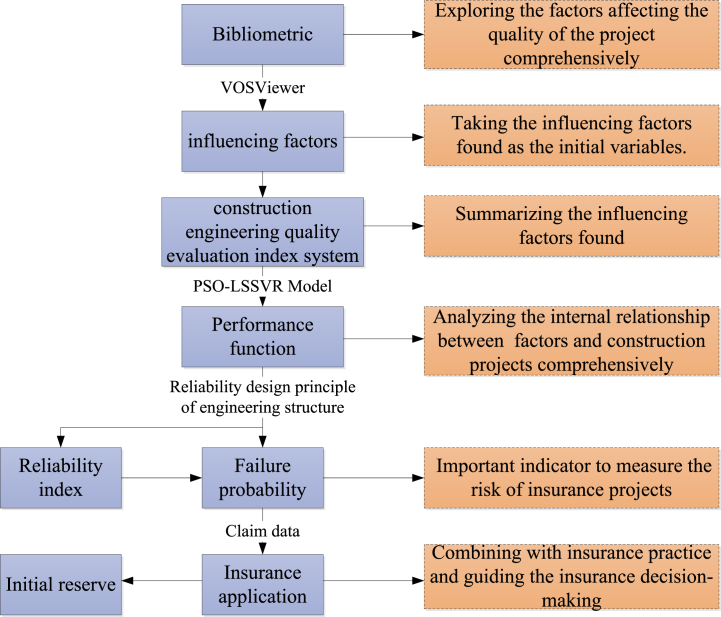
The structure and process of the paper.

From the perspective of insurance companies, insurance claims and the profitability of insurance projects are the main factors considered by insurance companies. The failure probability of construction projects is an important tool for insurance companies to make decisions from the perspective of insurance companies’ profits. Therefore, the failure probability of construction projects is an index that belongs to the insurance field. And the failure probability does not reflect the quality of construction projects, but is linked to the risk of insurance and the initial reserve. From the perspective of insurance companies, this paper makes an actuarial analysis of the insurance risk of IDI projects. The failure probability of construction projects is closely related to the claims of insurance projects. Therefore, it is necessary to analyze the failure probability of construction projects and explore the probability of possible problems in construction projects, which will help insurance companies better grasp project risks and formulate corresponding strategies, so as to better implement IDI. The overall structure and process of the paper are shown in Fig. 2.

## 2 Factor analysis and index system construction of inherent defect in construction engineering

### 2.1 Bibliometric method of inherent defect influencing factors

**Fig. 3:**
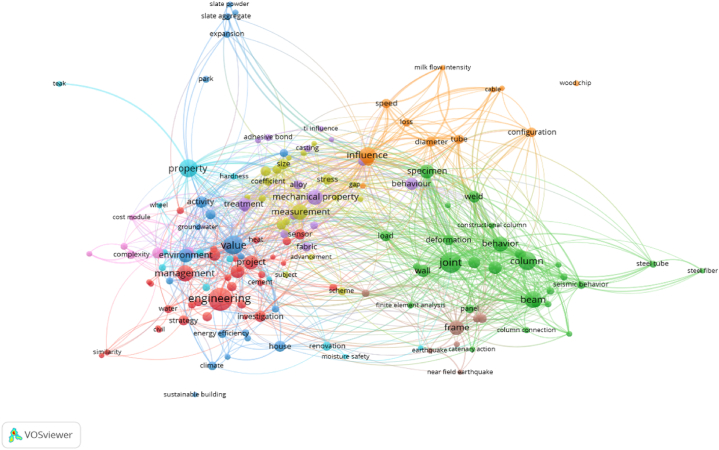
Knowledge map of quality influencing factors in construction period of construction engineering.

**Fig. 4:**
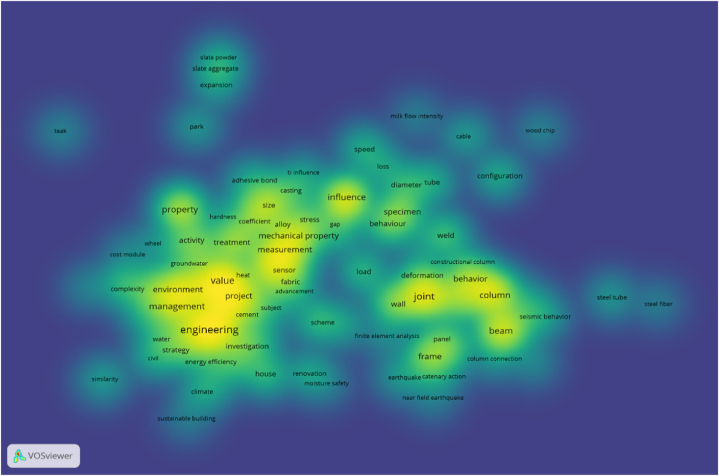
Density map of quality influencing factors in construction period of construction engineering.

To better explore the factors related to the quality of construction projects, this paper selected 500 papers with themes of ' construction quality ' and ' factor '. By extracting key information such as the title and abstract of the paper, VOSviewer software was used to draw a knowledge map of the factors influencing construction engineering quality. The mind and density maps obtained using the bibliometric method are shown in Fig. 3, Fig. 4.

As shown in Fig. 3, Fig. 4, the research hotspots for the quality and influencing factors of construction projects during the construction period can be roughly divided into five clusters identified by different colours. The red, pink and dark blue parts at the lower left are integrated, indicating that the three are closely related and can be analyzed together. It can be seen from the density map that the keywords in this section include ‘environment’, ‘management’ and ‘value’ in addition to search terms such as ‘engineering’ and ‘project’. Among these, ‘environment’ is mostly related to the natural environment, and ‘management’ and ‘value’ are mostly related to methods and means. Therefore, this cluster can be defined as an ‘environment and management method'.

Similarly, the green and brown parts of the lower-right part of Fig. 3 are also fused with each other; thus, they can be analyzed as a cluster. It can be seen from Fig. 4 that the keywords of the cluster mainly have two parts. One part included keywords such as ‘joint’ and ‘behavior’, which are mainly related to human behaviour. The other part included keywords such as ‘column’, ‘beam’ and ‘frame’, which are often related to construction methods of construction projects. Therefore, this cluster can be defined as ' member factors and construction methods'.

Similarly, the light-blue part of the upper left part is mainly around the keyword ‘property’, which can be defined as ‘material properties'. The purple and yellow parts in the middle are mainly around the keywords ‘mechanical property’ and ‘measurement’, ‘treatment’, and the introduction is mainly about the mechanical equipment and the corresponding coping methods of mechanical equipment failure, which can be defined as ‘mechanical equipment’. Except for the keyword ‘influence’ used in the search, the main keywords of the orange content on the upper right include keywords such as ‘diameter’, ‘tube’ and ‘configuration’, and the cluster can be defined as ‘external conditions'.

**Fig. 5:**
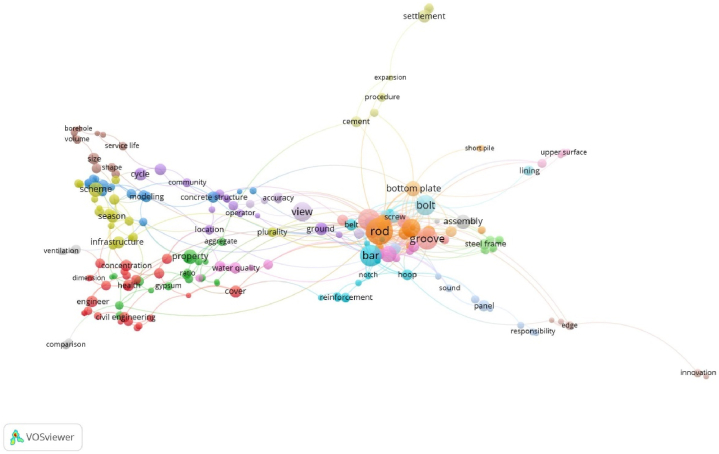
Knowledge map of quality influencing factors in construction period of operation engineering.

**Fig. 6:**
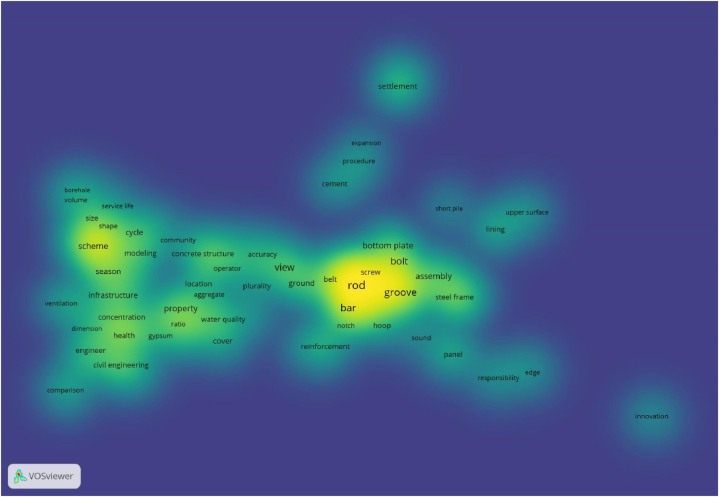
Density map of quality influencing factors in construction period of operation engineering.

Since IDI is related to the quality management of construction projects during operation, the same bibliometric analysis is used to analyze the quality of construction projects during operation. 176 papers with the topic ‘construction quality factor’ and the title ‘operation’ were selected in web of science. The mind map and density map obtained by the bibliometric method are shown in Fig. 5, Fig. 6 respectively.

As shown in Fig. 5, Fig. 6, the research results on the quality and factors influencing the construction project operation period can be roughly divided into two clusters. The left part is centred on ‘infrastructure’, and it can be seen from the density map that the keywords in this part include ‘season’, ‘health’ and ‘scheme’, in addition to search terms such as ‘civil engineering’ and ‘engineering’. These keywords are mostly related to the natural environment and engineering matters that are susceptible to the natural environment, so the cluster can be defined as ‘natural environmental factors'.

As shown in Fig. 5, the right-hand side can be analyzed as a cluster. It can be seen from Fig. 6 that the keywords of the cluster are centred on ‘rod’, and also include ‘groove’, ‘bolt’, ‘assembly’, ‘steel frame’ and other keywords. Most of these factors are related to building structure and architectural form; Therefore, this cluster can be defined as ‘construction method and structure’. As these keywords often appear as research objects or topics in the process of literature summarization, it is necessary to further examine whether such keywords are related to inherent defects.

### 2.2 Summary of inherent defect influencing factors

From the knowledge map of research on the influencing factors of construction engineering quality, it can be observed that the factors involved in the link of construction engineering quality can be roughly divided into several parts, such as the environment, method, human factors, materials, mechanical equipment and external conditions, which coincides with the idea of quality management in engineering management. In quality management, the factors that affect quality are summarized as the ‘4M1E principle’, which refers to Man, Machine, Material, Method and Environment [33].

Among them, people comprise the main body of production and operation activities, as well as decision-makers, managers, and project construction operators. The quality of personnel directly and indirectly affect the quality of a project to varying degrees [34]. Many equipment factors directly affect the quality of the project use function, such as product quality, equipment type, and equipment performance [35]. Engineering materials refer to all types of building materials, components and semi-finished products that constitute an engineering entity, which is the material condition of engineering construction, which will directly affects the structural stiffness and strength of the construction project [36]. In engineering construction, the rationality of the construction scheme, advancement of the construction technology and correctness of the construction operation have a significant impact on the quality of the project, which means that the use of new technologies, processes and methods to continuously improve the level of technology is an important factor in ensuring stable improvement of engineering quality [37]. Environmental conditions refer to the environmental factors that play an important role in the quality characteristics of the project, including the technical environment, working environment, management environment and surrounding environment of the project. This means that strengthening the management of environmental conditions, supplemented by necessary measures, is an important guarantee to control the impact of environmental conditions on project quality [38].

By sorting the factors influencing inherent defects in different aspects, it can be seen that the factors affecting quality in quality management are summarized by ‘4M1E principle’. However, the quality of personnel, mechanical equipment, engineering materials, construction methods and other factors are largely directly related to the qualifications of construction project participants [37,39,40]. Therefore, the qualifications of the participating parties can largely include factors such as personnel quality, mechanical equipment, engineering materials and construction methods.

In addition, the environmental impact of the project construction period on inherent defects was mainly concentrated in the policy environment. From the perspective of the impact of the policy environment, the social development of the project location and the importance of project supervision largely depend on the development of the local construction market [41,42]. The overall scale of the construction industry market is demonstrated by the total amount of real estate investments. Owing to the different volumes in different cities, this paper uses the total investment per capita of real estate for investigation and evaluation. Moreover, the number of local patent authorizations can represent the level of local technological innovation to a certain extent, which may also impact the quality of construction projects [43,44]. In addition, according to Western economics, any free market can be analyzed from both the supply and demand perspectives. On the supply side, the development of the local construction market can be evaluated and estimated based on the proportion of construction workers. On the demand side, the buyers’ purchase area can effectively reflect the development of the construction industry, which can be replaced by the per capita sales area of commercial housing.

Because the specific construction form and structure involved are often determined in the design stage of the construction project, they will not change according to the emergence of IDI. Therefore, the specific construction form and structure involved cannot be included in the index system of the influencing factors of inherent defects. Natural environmental factors primarily influence the construction engineering operation period. From the perspective of natural environmental factors, climatic factors such as temperature, wind speed, temperature difference and humidity may affect the quality of construction projects [40]. In addition, precipitation may lead to leakage in construction projects and sunshine can easily cause cracks, which are important components of construction quality defects [45]. Therefore, average precipitation and sunshine are also natural environmental factors for analyzing the quality defects of construction projects.

### 2.3 Construction of construction engineering quality evaluation index system

Based on a bibliometric analysis of the influencing factors, the member, natural environmental and policy environmental factors were determined as the criterion layers, and the corresponding indicators ware determined. The evaluation index system for inherent defects is presented in Table 1.

**Table 1:** Construction of the engineering quality evaluation index system.

Criterion layer	Influencing factors	Abbreviation	References
Member factor	Owner enterprise score	OES	[34,37]
	Construction enterprise score	CES	[34,39]
	Supervision enterprise score	SES	[34,40]
Natural environmental factor	Average temperature	AT	[38,40]
	Average wind velocity	AWV	[38,40]
	Average temperature variation	ATV	[38,40]
	Average humidity	AH	[38,40]
	Average precipitation	AP	[38,45]
	Average sunshine	AS	[38,45]
Policy environmental factor	Per-capita real estate investment	PREI	[41,42]
	Patent number	PN	[43,44]
	Proportion of construction workers	PCW	[44]
	Per-capita commercial housing sales area	PCHSA	[44]

## 3 Determination of random variable distribution based on PSO-LSSVR model

Based on the construction project quality defect index evaluation system, the factors affecting inherent defects can be summarized as member, natural environmental and policy environmental factors. The member factor can be expressed as $X_{1},X_{2},X_{3}$, which represents the score of owner, construction and supervision enterprise respectively; the natural environment factor can be expressed as $Y_{1},Y_{2},...,Y_{6}$, which represents average temperature, average wind velocity, average temperature variation, average humidity, average precipitation and average sunshine respectively; the policy environment factor can be expressed as $Z_{1},Z_{2},Z_{3},Z_{4}$, which represents per-capita real estate investment, patent number, proportion of construction workers and the policy environment factors can be expressed as, which represent four indicators of per capita investment in real estate, the number of patents granted, the proportion of construction workers and per-capita commercial housing sales area respectively. The risk function of inherent defects in construction quality can be derived as equation (1):

(1) $$G=g\left(X_{1},X_{2},X_{3},Y_{1},Y_{2},...,Y_{6},Z_{1},Z_{2},Z_{3},Z_{4}\right)$$

### 3.1 Model construction

#### 3.1.1 Least squares Support Vector Regression(LSSVR)

Support Vector Regression (SVR) is a nonlinear regression model based on Support Vector Machine (SVM) and statistical theory [46]. The regression problem is transformed into an optimization problem, and the kernel function is used to map the input features to a high-dimensional feature space, and then the optimal hyperplane is found in the feature space. The advantages and disadvantages of SVR model and alternative prediction models are shown in Table 2.

**Table 2:** The advantages and disadvantages of SVR model and alternative prediction models.

Prediction models	Advantage	Disadvantage	Reference
Multiple Linear Regression	1 The calculation is simple and can quickly analyze the relationship between variables. 2 The results intuitively show the influence degree and relationship of variables.	1 Can not accurately deal with the problem of collinearity. 2 The prediction accuracy will decrease for data sets with small sample size.	[47,48]
Conditional Inference Trees	1 It is easy to understand and explain, and can deal with nonlinear relationships and interaction effects. 2 Robust to missing data and outliers.	1 It is easy to over-fit and sensitive to noise. 2 Not suitable for dealing with continuous variables.	[49]
Random Forests	1 Classification and numerical features can be processed simultaneously. 2 The stability is strong, and the whole algorithm will not be affected too much by the new data points.	1 Noise processing is not good, and may produce the phenomenon of fitting. 2 The algorithm is complex and the computational cost is higher.	[47]
Neural Networks	1 It has strong robustness and fault tolerance. 2 It has strong information integration ability and can well coordinate the relationship of multiple input information.	1 The output results are uncertain and lack of explanation. 2 The data fitting ability for small sample size is poor.	[48]
Support Vector Regression	1 It is suitable for small sample analysis because the law of large numbers is not involved. 2 It has good robustness and is not easy to receive the influence of outliers. 3 The nonlinear and high dimensional data can be handled for the kernel function.	1 The complexity of the model leads to a high amount of calculation. 2 It takes a long training time.	[50]

It can be seen from Table 2 that the SVR model has the advantages of strong nonlinear analysis ability and can deal with outliers and high-dimensional data. Although there are problems of high computational complexity and long training time in SVR model, the SVR model is worth being selected to fit the function regression of engineering quality. The basic operation process of the model is as follows.

First, SVR maps the training data, the actual problem, from a low-dimensional space to a high-dimensional feature space by a nonlinear mapping function, and then performs linear regression. The same effect is obtained in the high-dimensional feature space as the nonlinear regression in the original space, which is based on the following principle: given a set of training sample points: $S=\{\left(x_{k},y_{k}\right)|k=1,2,...,n\}$, where $x_{k}\in R^{n}$ is the input vector and $y_{k}\in R$ is the output vector, the regression problem is to find a single-valued function from the input space $R^{n}$ to the output space R, as shown in equation (2):

(2) $$f:R^{n}\to R,s.t.\;f\left(x\right)=y$$

Since there is a certain error between the function found by the regression and the original output data, at a certain level of error allowed ε, the ε-insensitive loss function can be defined as equation (3):

(3) $${\left|y-f\left(x\right)\right|}_{\varepsilon }=\left\{ \begin{array}{c} 0,\left|y-f\left(x\right)\right|\leq \varepsilon \\ \left|y-f\left(x\right)\right|-\varepsilon ,otherwise \end{array}\right.$$

where f(x) is the regression function, y is the output data of the input data x, and ε is a parameter greater than zero and directly related to the accuracy of the estimated function. The purpose of machine learning is to obtain the optimal regression function f(x) so that the distance between the target value and f(x) is less than ε. To better reduce the fitting accuracy error so that it satisfies the accuracy requirement ε, the slack variable needs to be introduced to solve this problem:

(4) $$\left\{ \begin{array}{c} \min\frac{1}{2}\omega^{T}\omega +\gamma \sum_{k=1}^{n}\left(\xi_{k}+\xi_{k}^{*}\right)\\ s.t.\;\omega^{T}\varphi \left(x_{k}\right)+b-y_{k}\leq \varepsilon +\xi_{k}^{*}\\ y_{k}-\left[\omega^{T}\varphi \left(x_{k}\right)+b\right]\leq \varepsilon +\xi_{k}\\ \xi_{k},\xi_{k}^{*}\geq 0\left(k=1,2,...,n\right) \end{array}\right.$$

where γ is the regularization parameter and $\xi_{i},\xi_{i}^{*}$ is the slack variable, so that equation (4) can be transformed into equation (5):

(5) $$\left\{ \begin{array}{c} \min\;Q\left(\alpha \right)=\frac{1}{2}\sum_{k=1}^{n}\left(\alpha_{k}-\alpha_{k}^{*}\right)\left(\alpha_{j}-\alpha_{j}^{*}\right)\left(x_{k},x_{j}\right)-\varepsilon \sum_{k=1}^{n}\left(\alpha_{k}+\alpha_{k}^{*}\right)+\sum_{k=1}^{n}y_{k}\left(\alpha_{k}-\alpha_{k}^{*}\right)\\ s.t.\;\sum_{k=1}^{n}\left(\alpha_{k}+\alpha_{k}^{*}\right)=0\\ \alpha_{k},\alpha_{k}^{*}\in \left[0,C\right] \end{array}\right.$$

Then, the nonlinear regression function with the inner product as the kernel function can be expressed as equation (6):

(6) $$f\left(x\right)=\sum_{k=1}^{n}\left(\alpha_{k}-\alpha_{k}^{*}\right)K\left(x_{k},x\right)+b$$

On the basis of the SVR model, the least squares method can be introduced to construct the LSSVR model to guarantee the accuracy of the model. The LSSVR model not only retains the advantages of SVR model, such as good generalization performance and ability to deal with nonlinear problems, but also has the characteristics of fast training speed, which makes it widely used in the fields of modelling, prediction and pattern recognition. For the same training sample points: $S=\left\{\left(x_{k},y_{k}\right)|k=1,2,...,n\right\}$, where $x_{k}\in R^{n}$ is the input vector and $y_{k}\in R^{n}$ is the output vector. Through the nonlinear mapping φ(·), the samples from the original space $R^{n}$ samples are mapped to the feature space $\varphi \left(x_{k}\right)$ [50]. The optimal decision function is defined as equation (7):

(7) $$y\left(x\right)=\omega^{T}\cdot \varphi \left(x\right)+b$$

where φ(x) is the kernel space mapping function, ω is a weighting vector, and b is a constant. The coefficients ω and b are usually obtained by minimizing an upper bound on the generalization error.

According to the principle of structural risk minimization, the regression problem can be transformed into an optimization problem with constraints such that the objective function and constraints can be expressed as equation (8):

$$\min_{\omega ,b,e}\left(\omega ,e\right)=\frac{1}{2}\omega^{T}\omega +\frac{1}{2}\gamma \sum_{k=1}^{n}e_{k}^{2}$$

(8) $$s.t.\;y_{k}=\omega^{T}\varphi \left(x_{k}\right)+b+e_{k}$$

where k=1,2,...,n, γis the regularization parameter and $e_{k}$ is the slack variable.

In order to solve this optimization problem, a Lagrangian function can be introduced to solve it, which is defined as equation (9):

(9) $$L\left(\omega ,b,e,\alpha \right)=\varphi \left(\omega ,e\right)-\sum_{k=1}^{n}\left\{\alpha_{k}\left[\omega^{T}\cdot \varphi \left(x\right)+b+e_{k}-y_{k}\right]\right\}$$

where $\alpha_{k}$ is the Lagrangian multiplier. According to the Karush-Kuhn-Tucker condition, the optimality is conditioned by

(10) $$\left\{ \begin{array}{l} \omega =\sum_{k=1}^{n}\alpha_{k}\varphi \left(x_{k}\right)\\ \sum_{k=1}^{n}\alpha_{k}=0\\ \alpha_{k}=e_{k}\gamma \\ \omega^{T}\cdot \varphi \left(x_{k}\right)+b+e_{k}-y_{k}=0 \end{array}\right.$$

Equation (10) can be converted into a linear equation by matrix expression as equation (11):

(11) $$\left[\begin{array}{cccc} 0 & 1 & \cdots & 1\\ 1 & K\left(x_{1},y_{1}\right)+\frac{1}{\gamma } & \cdots & K\left(x_{1},y_{1}\right)\\ \vdots & \vdots & \ddots & \vdots \\ 1 & K\left(x_{1},y_{1}\right) & \cdots & K\left(x_{1},y_{1}\right)+\frac{1}{\gamma } \end{array}\right]\left[\begin{array}{c} b\\ \alpha_{1}\\ \vdots \\ \alpha_{l} \end{array}\right]=\left[\begin{array}{c} 0\\ y_{1}\\ \vdots \\ y_{l} \end{array}\right]$$

This allows the values of α and b to be obtained by fitting the function, which is expressed as equation (12):

(12) $$f\left(x\right)=\sum_{k=1}^{l}\alpha_{k}K\left(x,x_{i}\right)+b$$

where $K\left(x,x_{i}\right)=\varphi {\left(x\right)}^{T}\cdot \varphi \left(x_{i}\right)$ is a kernel function that represents a nonlinear mapping from a low-dimensional space to a high-dimensional space.

Due to the kernel function, the mapping function and the feature space are basically in one-to-one correspondence. The kernel function $K\left(x_{i},x_{j}\right)$ determines the mapping function φ(x) and the feature space F. It helps the LSSVR model to better handle nonlinear data and maintain internal linear relationships in high-dimensional space. The most common kernel functions include five types of linear functions, polynomial functions, Fourier functions, neural network S-functions and radial basis functions, among which the simple expressions of radial basis functions help to handle large amount of input data easily, while having good smoothness, radial symmetry, and good resolution for theoretical analysis. Therefore, radial basis function is chosen as the type of kernel function in this paper, and the kernel function formula of radial basis function is as equation (13):

(13) $$K\left(x,x_{i}\right)=\exp\left(\frac{-{\left\|x-x_{i}\right\|}^{2}}{2\sigma^{2}}\right)$$

where x is the m-dimensional input vector, $x_{i}$ is the center of the i-th radial basis function, $\sigma^{2}$ is the length of the kernel function, and $\left\|x-x_{i}\right\|$ is the van of the vector to represent the distance between x and $x_{i}$.

#### 3.1.2 Particle Swarm Optimization(PSO)

The PSO that simulates the hunting activities of birds and fish is a stochastic search algorithm [51] which is capable of solving the global optimal solution. The PSO model can further solve the problems of complex calculation and long training time of SVR model by improving the training speed of the model. PSO is initialized with a random population containing m particles, where $X=\left\{X_{1},X_{2},...,X_{m}\right\}$. Each particle is a point in the D-dimensional space and a feasible solution in the solution space, respectively. The particles change their positions by randomly shifting in the space until the optimal solution is reached. First set the position of the i-th particle as $X_{i}=\left\{x_{i1},x_{i2},...,x_{id}\right\}\left(i=1,2,...,m\right)$, and also set the velocity of the i-th particle as $V_{i}=\left\{v_{i1},v_{i2},...,v_{id}\right\}\left(i=1,2,...,m\right)$, $P_{best}\left(i\right)$ is the local optimal solution and $G_{best}$ is the overall optimal position [44]. The position of the i-th particle in the k-th iteration is calculated as follows:

(14) $$x_{id}\left(k+1\right)=x_{id}\left(k\right)+v_{id}\left(k+1\right)$$

The velocity of the i-th particle in the k-th iteration is calculated as follows:

(15) $$v_{id}\left(k+1\right)=w\cdot v_{id}\left(k\right)+c_{1}r_{1}\cdot \left(P_{best}\left(i\right)-x_{id}\left(k\right)\right)-c_{2}r_{2}\cdot \left(G_{best}-x_{id}\left(k\right)\right)$$

where $r_{1}$ and $r_{2}$ are two random numbers in the range [0,1], $c_{1}=c_{2}=2$ is the acceleration coefficient, and ω is the inertia weighting factor, as determined by Equation (16).

(16) $$w=w_{\max}-\left(w_{\max}-w_{\min}\right)\frac{n_{i}}{n_{\max}}$$

The Steps of Particle Swarm Optimization are as follows.

Step 1 Set the parameters of the PSO algorithm, such as particle swarm size, inertial weight factor, learning factor, and random number.

Step 2 Randomly initialize the velocity and position of the particles.

Step 3 Compute the fitness of the ith particle and obtain the local best position and the global best position.

Step 4 Update the local best position and the global best position.

Step 5 Update the velocity and position of the particles via Eq. (14) and Eq. (15).

Step 6 If the maximum number of iterations is achieved or the optimum solution is found, then we may get the global best position; otherwise return to Step 3.

The different parameters used as input combinations in the LSSVR model can be modeled to produce various prediction accuracies. Therefore, SO-LSSVR model should be given more importance because it allows the selection of optimized parameters in the modeling. The PSO algorithm optimizes the kernel parameter $\sigma^{2}$ and the regularization parameter γ in the LSSVM, which can improve the prediction accuracy and reduce the uncertainty and stochasticity of the model.

**Fig. 7:**
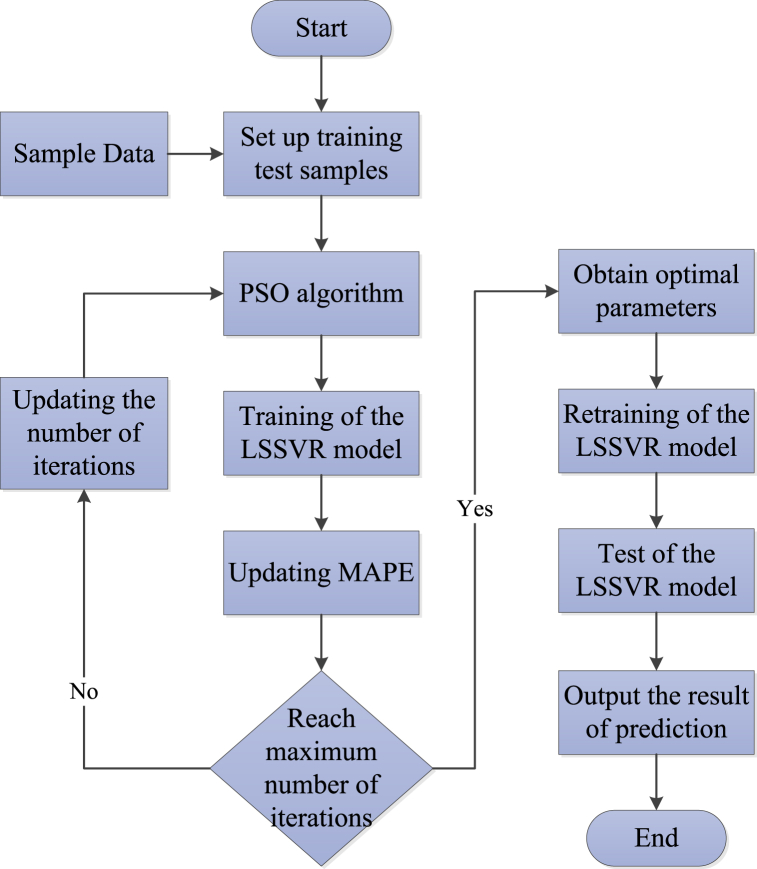
The operation mechanism of the PSO-LSSVR model.

The operation mechanism of PSO-LSSVR model is shown in Fig. 7.

### 3.2 Construction quality inherent defects performance function fitting

#### 3.2.1 Data collection and preprocessing

To further determine the risk function of inherent defects in construction project quality, it is necessary to organize the construction information and the quality of construction projects. Therefore, in this paper, nearly 100 pre-evaluation data points from the same professional quality inspection agency in 12 cities in China were collected to identify the factors which affect the quality of construction projects. Proportion of construction workers belongs to urban macro data, so it is completely obtained from China City Statistical Yearbook by China National Bureau of Statistics. For other indicators, the data can be obtained from the quality report of the project. The data not reflected in the quality report of the project is supplemented by the data of located city from China City Statistical Yearbook by China National Bureau of Statistics and the official website of China Meteorological Data Service Center. The descriptive statistical analysis of the data is shown in Table 3.

**Table 3:** The descriptive statistical analysis of the data.

Project Classification		Number of project	Frequency of project
Project Location	Hefei, Anhui province	18	18.750%
	Chongqing	36	37.500%
	Changzhou, Jiangsu province	1	1.042%
	Qinzhou, Guangxi province	2	2.083%
	Huizhou, Guangdong province	2	2.083%
	Huzhou, Zhejiang province	1	1.042%
	Jiangmen, Guangdong province	1	1.042%
	Guangzhou, Guangdong province	1	1.042%
	Shenzhen, Guangdong province	24	25.000%
	Fuzhou, Fujian province	1	1.042%
	Xiamen, Fujian province	7	7.292%
	Tianjin	2	2.083%
Project Types	Residential building	75	78.125%
	Public building	21	21.875%

Based on the descriptive statistical analysis of the original data, the multicollinearity of the data can be analyzed by Variance Inflation Factor (hereinafter referred to VIF) test. The VIF test is implemented by Stata 16, and the VIF test values are shown in Table 4.

**Table 4:** Result of VIF test.

Variable	VIF	1/VIF
OES	1.14	0.873887
CES	1.39	0.721874
SES	1.81	0.551956
AT	9.53	0.104895
AWV	8.97	0.111435
ATV	8.73	0.114556
AH	6.30	0.158814
AP	9.76	0.102431
AS	8.11	0.123232
PREI	9.62	0.103987
PN	4.36	0.229594
PCW	9.76	0.102450
PCHSA	1.84	0.544279
Mean VIF	6.26	

It can be seen from Table 4 that the VIF test values of the original data and its average are less than 10, which indicates that the multicollinearity of the data is not significant. Therefore, the data can be used for fitting analysis, and the variables selected by the bibliometric analysis are initial variables.

Because the initial data have different sample value intervals, which may affect the accuracy of function fitting, the initial data must be normalized first. According to the central limit theorem, when the number of samples is sufficiently large, the distribution of a series of samples with independent and identical distributions gradually converges to the normal distribution [52]. Therefore, in this paper, the standardization method of the standard normal distribution was chosen to transform the initial samples. The formula for the initial data standardization is as equation (17):

(17) $$\overset{\wedge }{x}=\frac{x-\bar{x}}{sd\left(x\right)}$$

where $\bar{x}$ represents the sample mean, sd(x) represents the sample standard deviation, x represents the sample value before standardization, and $\overset{\wedge }{x}$ represents the sample value after standardization.

#### 3.2.2 Performance function fitting

To better measure the effect of function fitting, a multiple regression model for performance function fitting was used first. Multiple regression is the most commonly used method of function fitting and which quantitatively portrays the interrelationship between a response variable and multiple independent variables using regression equations [53]. The multiple regression fitting of the standardized sample data yields.

**Fig. 8:**
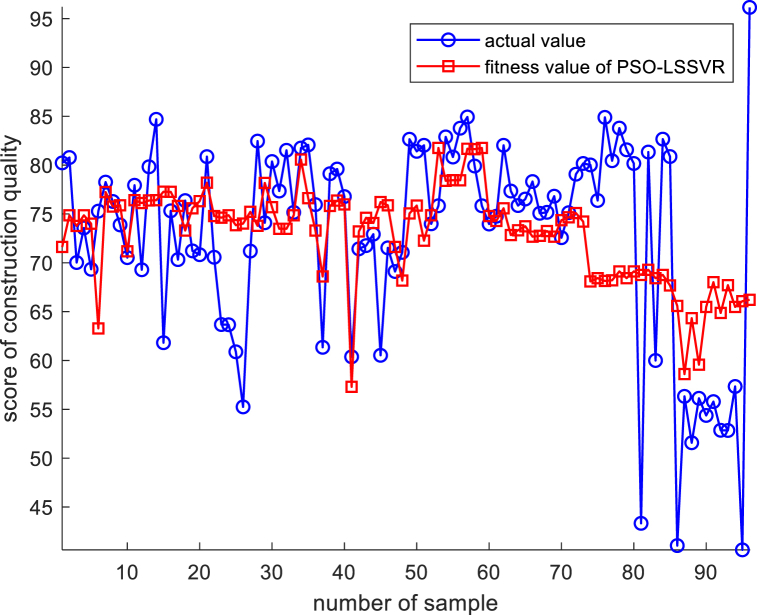
Multiple regression model performance function fitting effect.

**Fig. 9:**
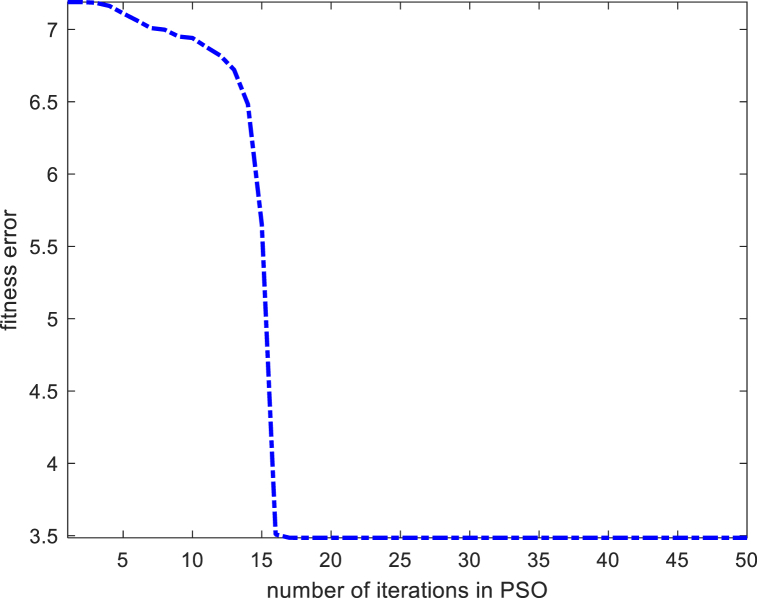
Iterative effect of PSO model.

Fig. 8 shows the fitting effect for the inherent defect performance function of construction quality under the multiple regression model, indicating that there are many sample points with deviations in the fitting effect. Subsequently, this paper then uses the PSO-LSSVR model for performance function fitting, and on the basis of standardizing the initial data, the standardized initial data are brought into the LSSVR model for regression analysis, and the PSO model is used for iteration optimization, with the maximum number of iterations set to 50, and the relationship between the number of model iterations and accuracy is shown in Fig. 9.

**Fig. 10:**
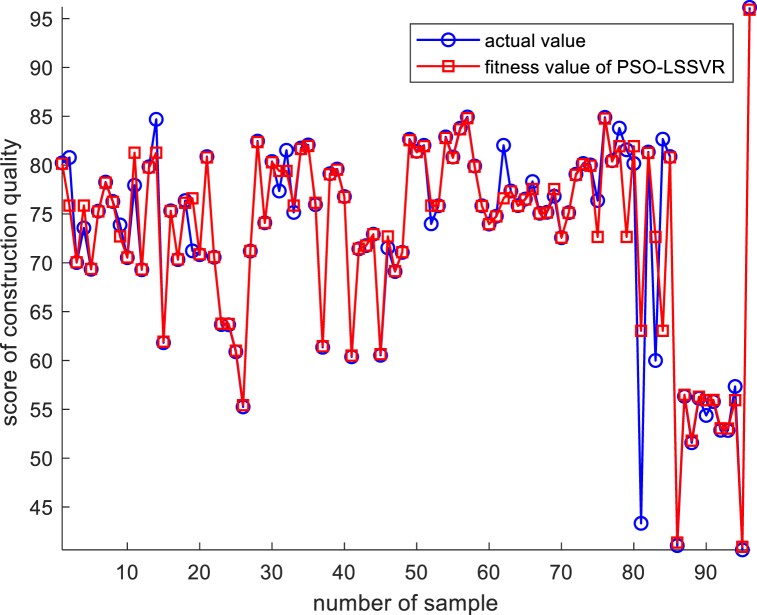
PSO-LSSVR model performance function fitting effect.

It can be seen that after the optimization of the PSO model, the model error is controlled, indicating that the optimization of the PSO model has obvious effects. The sample fitting plots obtained based on the PSO-optimized LSSVR model are shown in Fig. 10.

From the above figure, it can be seen that the PSO-LSSVR model has better function fitting compared to the fit of multiple regression, and the vast majority of the sample and fitted points overlap. To further demonstrate the effectiveness of the model, indicators for evaluating model error ware introduced in this paper, and the degrees of fit of the two models are shown in Table 5.

**Table 5:** Comparison of errors between PSO-LSSVR model and multiple regression model.

error index	MAPE	RMSE	MSE	R^2^
PSO-LSSVR model	1.6884	3.5034	12.2736	0.95265
Multiple regression model	9.7668	9.2286	85.1668	0.87528

MAPE refers to the mean absolute percentage error, which is a relative measure that uses absolute values to prevent positive and negative errors from cancelling each other out; RMSE refers to the root mean square error, which is the square root of the ratio of the square of the deviation of the predicted value from the true value to the number of observations n; and MSE refers to the mean square error, which is the expected value of the square of the difference between the estimated value of a parameter and the true value of that parameter. The larger the values of MAPE, RMSE and MSE, the larger the error between the fitted and actual values, and the lower the accuracy of the model. R^2^ refers to the goodness of fit, which is the degree of fit of the regression line to the observed value. The closer the value of R^2^ is to 1, the better the fit of the regression function to the observed value; conversely, the smaller the value of R^2^, the worse the fit of the regression function to the observed value [53]. The PSO-LSSVR model fit is much better than that of the multiple regression analysis; therefore, the performance function fitted by the PSO-LSSVR model was used for further analysis in this paper.

#### 3.2.3 Functional regression analysis

Based on the fitting of the PSO-LSSVR model to the inherent defect performance function of the quality of construction work, the regression coefficients of the function fitting based on the PSO-LSSVR model were derived, and the fitting coefficients were processed using the absolute values, as shown in Table 6.

**Table 6:** PSO_LSSVR fitting coefficients.

Criterion layer	The abbreviation of influencing factors	Fitting coefficients
Member factor	OES	2.945
	CES	0.362
	SES	0.496
Natural environmental factor	AT	8.728
	AWV	8.425
	ATV	3.906
	AH	13.138
	AP	21.606
	AS	8.629
Policy environmental factor	PREI	4.506
	PN	11.207
	PCW	11.977
	PCHSA	0.112
–	b	72.872

Table 6 shows the approximate influence of different influencing factors on inherent defects in construction quality. From the regression coefficients, the mean absolute value of the regression coefficient corresponding to the personnel factor is small, indicating that the degree of influence of the personnel factor is not as high as that of the natural and policy environment factors. It is related to the identification of enterprise qualification and the improvement of hierarchical management system. The perfect enterprise qualification management system and effective project access conditions have prevented a large number of enterprises with lower qualifications from entering the market. Therefore, the score gap of the personnel factor part is relatively small, resulting in a smaller absolute value of the regression coefficient. Among the personnel factors, the absolute value of the regression coefficient of the construction unit is the largest, which indicates that the construction unit plays a larger role in the quality of construction projects, which is consistent with the primary responsibility of the construction unit as stipulated by the law. Similarly, in the natural environment factors, the absolute value of the regression coefficient of the average humidity and average precipitation is greater than that of the other factors, indicating that humidity and precipitation have a greater degree of influence on the quality of the project's inherent defects, which is also consistent with the actual waterproofing and insulation projects exposed to the most problems. Among the policy environment factors, the absolute value of the regression coefficient of the patents number and the proportion of construction workers is higher, which is due to the fact that China ‘s construction industry is currently undergoing a period of transition from labor-intensive to technology-intensive. Therefore, the patents number and the proportion of construction workers have a great impact on the development of the construction industry. Correspondingly, the absolute value of the regression coefficient of Per-capita commercial housing sales area is low, which indicates that the quality problems of construction projects are more caused by the supply side, and the demand side has a relatively small impact on the probability of construction quality problems.

## 4 The quality of construction projects inherent defects failure probability and reliability analysis

### 4.1 Failure probability and reliability analysis model construction

Because the preceding section has analyzed the engineering quality inherent defects of each influencing factor, in the case of large samples, the estimation of the approximate normal distribution as well as the standardization can be carried out, and the performance function is fitted linearly in this paper, which can be expressed as

(18) $$Z=g_{x}\left(X_{1},X_{2},...,X_{n}\right)=\alpha_{0}+\sum_{i=1}^{n}\alpha_{i}X_{i}$$

where $\alpha_{i}\left(i=0,1,...,n\right)$ is a constant.

To better reflect the geometric significance of reliable indicators in the standard normal coordinate system, it is necessary to transform the normally distributed variables into variables of standard normal distribution by normalization as equation (19):

(19) $$Y_{i}=\frac{X_{i}-\mu_{x_{i}}}{\sigma_{x_{i}}}\left(i=1,2,..,n\right)$$

The original performance function can therefore be expressed as equation (20):

(20) $$Z=g_{Y}\left(Y_{1},Y_{2},...,Y_{n}\right)=\alpha_{0}+\sum_{i=1}^{n}\alpha_{i}\left(\mu_{x_{i}}+\sigma_{x_{i}}Y_{i}\right)=\alpha_{0}+\sum_{i=1}^{n}\alpha_{i}\mu_{x_{i}}+\sum_{i=1}^{n}\alpha_{i}\sigma_{x_{i}}Y_{i}$$

From this, the mean and standard deviation of the performance function can be obtained as equations (21), (22):

(21) $$\mu_{Z}=\alpha_{0}+\sum_{i=1}^{n}\alpha_{i}\mu_{x_{i}}$$

(22) $$\sigma_{Z}=\sqrt{\sum_{i=1}^{n}\alpha_{i}^{2}\sigma_{x_{i}}^{2}}$$

This is similar to the definition of structural reliability indicators in reference to engineering to clarify the quality of construction work inherent defects reliability indicators as follows:

(23) $$\beta =\frac{\mu_{Z}}{\sigma_{Z}}=\frac{\alpha_{0}+\sum_{i=1}^{n}\alpha_{i}\mu_{x_{i}}}{\sqrt{\sum_{i=1}^{n}\alpha_{i}^{2}\sigma_{x_{i}}^{2}}}$$

Based on the reliability index of inherent defects in construction quality, the failure probability can be further determined. Because the variables related to inherent defects in construction quality can be approximated as obeying the normal distribution and standardized, the formula for failure probability is determined as $p_{Z}=\Phi \left(-\beta \right)$ [54].

The model was then tested from a geometric perspective. First, let the performance function Z=0, denote the limit state, and divide $-\sqrt{\sum_{i=1}^{n}\alpha_{i}^{2}\sigma_{x_{i}}^{2}}$ equally on both sides of the equation to obtain equation (24):

(24) $$\sum_{i=1}^{n}\frac{-\alpha_{i}\sigma_{x_{i}}Y_{i}}{\sqrt{\sum_{j=1}^{n}\alpha_{j}^{2}\sigma_{x_{j}}^{2}}}-\frac{\alpha_{0}+\sum_{j=1}^{n}\alpha_{j}\mu_{x_{j}}}{\sqrt{\sum_{j=1}^{n}\alpha_{j}^{2}\sigma_{x_{j}}^{2}}}=0$$

Bringing in the formula of reliability index yields can obtain equation (25):

(25) $$\sum_{i=1}^{n}\frac{-\alpha_{i}\sigma_{x_{i}}Y_{i}}{\sqrt{\sum_{j=1}^{n}\alpha_{j}^{2}\sigma_{x_{j}}^{2}}}-\beta =0$$

It can be transformed by cosine function and equation (26) can be obtained:

(26) $$\alpha_{Y_{i}}=\cos\;\theta_{Y_{i}}=-\frac{\alpha_{i}\sigma_{x_{i}}}{\sqrt{\sum_{j=1}^{n}\alpha_{j}^{2}\sigma_{x_{j}}^{2}}}\left(i=1,2,...,n\right)$$

then equation (27) can be obtained:

(27) $$\sum_{i=1}^{n}\alpha_{Y_{i}}Y_{i}-\beta =\sum_{i=1}^{n}\cos\;\theta_{Y_{i}}Y_{i}-\beta =0$$

From the knowledge of analytic geometry, it can be seen that the equation is a normal equation, where $\cos\;\theta_{Y_{i}}$ is the cosine of the angle between the normal of the line and the coordinate axis, thus satisfying $\sum_{i=1}^{n}\alpha_{Y_{i}}^{2}=\sum_{i=1}^{n}\cos\;\theta_{Y_{i}}^{2}=1$, β is the shortest distance from the origin of the coordinates to the line. According to the geometric meaning of the reliability index, the foot of the vertical line from the origin of the coordinates to line is defined as the check point. Because the variables are pre-normalized, the coordinates of the check point before and after normalization are ${\left(x_{1}^{*},x_{2}^{*},...,x_{n}^{*}\right)}^{T}$ and ${\left(y_{1}^{*},y_{2}^{*},...,y_{n}^{*}\right)}^{T}$, respectively, and the relationship between them can be solved inversely according to the normalization formula as $x_{i}^{*}=\mu_{x_{i}}+\sigma_{x_{i}}y_{i}^{*}$ [54].

According to the geometric meaning of the reliability index, in the normalized coordinate system, the reliability index is related to the directional cosine of the linear normal as equation (28):

(28) $$y_{i}^{*}=\beta \alpha_{Y_{i}}=\beta \;\cos\;\theta_{Y_{i}}\left(i=1,2,...,n\right)$$

Converting them to the pre-standardized coordinate system and equation (29) can be obtained:

(29) $$x_{i}^{*}=\mu_{X_{i}}+\alpha_{X_{i}}\beta \sigma_{X_{i}}=\mu_{X_{i}}+\beta \sigma_{X_{i}}\;\cos\;\theta_{X_{i}}\left(i=1,2,...,n\right)$$

where $\alpha_{X_{i}}$ and $\cos\;\theta_{X_{i}}$ represent $\alpha_{Y_{i}}$ and $\cos\;\theta_{Y_{i}}$, respectively, as represented in the new coordinate system. The performance function $Z=g_{X}\left(x_{1}^{*},x_{2}^{*},...,x_{n}^{*}\right)=0$ can be easily verified by bringing the converted coordinates of the test point ${\left(x_{1}^{*},x_{2}^{*},...,x_{n}^{*}\right)}^{T}$ and the parameter equations $\alpha_{X_{i}}$ and $\cos\;\theta_{X_{i}}$ into the performance function, which means that the test point $x^{*}$ is a point on the limit state plane and the constructed reliability index model is valid.

### 4.2 Failure probability and reliability analysis based on PSO-LSSVR model

Based on the inherent defects failure probability and reliability analysis model constructed in the preceding section, this paper analyzes the performance function fitted by the PSO-LSSVR model. Because the variables are normalized and transformed when function regression fitting is performed, the performance function that conforms to Equation (18) is obtained as equation (30):

(30) $$Z=\alpha_{0}+\sum_{i=1}^{n}\alpha_{i}\mu_{x_{i}}+\sum_{i=1}^{n}\alpha_{i}\sigma_{x_{i}}Y_{i}$$

The parameters obtained from the calculations for each variable are listed in the following table.

The variable coefficient in Table 7 is the parameter fitted by the PSO-LSSVR model, and the significance of its absolute value has been analyzed in Table 6. Mean of variable and standard deviation of variable are obtained by statistical analysis based on pre-evaluation data. Both mean of variable and standard deviation of variable are the main part of constructing the performance function of construction engineering, and reflect the distribution of each influencing factor sample in the pre-evaluation data.

**Table 7:** Performance function coefficients.

Variable number	Variable abbreviation	Variable coefficient $\alpha_{i}$	Mean of variable $\mu_{x_{i}}$	Standard deviation of variable $\sigma_{x_{i}}$
0	b	72.872		
1	OES	2.945	87.4740	16.2739
2	CES	−0.362	94.7969	4.4418
3	SES	0.496	70.6771	8.9977
4	AT	−8.728	19.1655	2.9989
5	AWV	−8.425	4.4848	1.2064
6	ATV	−3.906	7.9262	1.0692
7	AH	−13.138	75.8303	2.8152
8	AP	21.606	3.7996	1.0070
9	AS	−8.629	4.3434	0.9066
10	PREI	−4.506	26502.7636	16914.9932
11	PN	−11.207	5.4899	3.4540
12	PCW	11.977	73848.7240	61071.0348
13	PCHSA	0.112	1.6452	0.4306

From the parameters of the performance function, the mean and standard deviation of the inherent defects performance function of the quality of construction works are $\mu_{Z}=764146.9316,\sigma_{Z}=735408.1837$, the results of which are introduced into Equation (23) as follows, and equation (31) can be obtained:

(31) $$\beta =\frac{\mu_{Z}}{\sigma_{Z}}=\frac{\alpha_{0}+\sum_{i=1}^{n}\alpha_{i}\mu_{x_{i}}}{\sqrt{\sum_{i=1}^{n}\alpha_{i}^{2}\sigma_{x_{i}}^{2}}}\approx 1.039$$

**Fig. 11:**
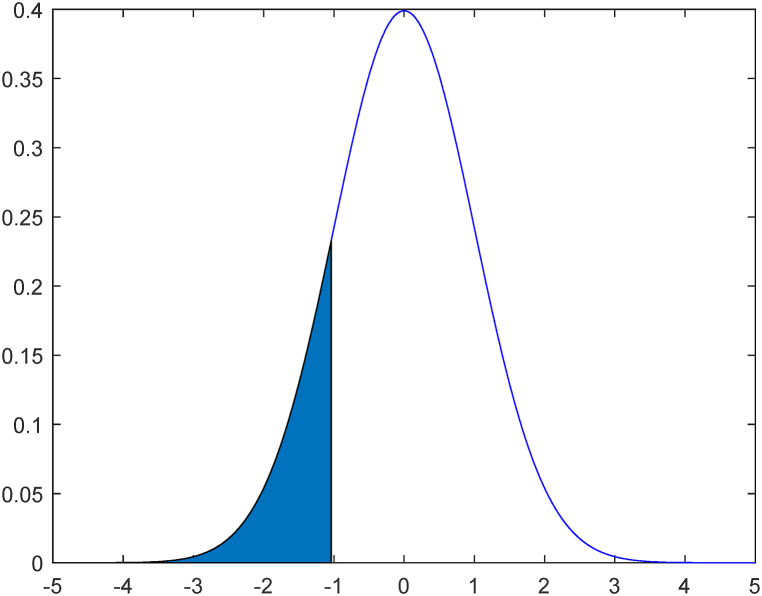
Construction quality inherent defects failure probability diagram.

Based on the obtained reliability index, the failure probability can be determined according to the function of the standard normal distribution, as shown in Fig. 11.

The proportion of the blue area in the left side of the figure to the whole curve area shows the failure probability of inherent defects in construction works. From the standard normal distribution calculation table, $p_{Z}=\Phi \left(-\beta \right)=0.1492$, and it is known that the failure probability of inherent defects in construction works is 0.1492. This indicates that according to the available statistics of inherent defects in construction quality, the probability of inherent defects in general construction works is 14.92%. To better cope with the risk of inherent defects that can occur in general construction projects, the quality of construction projects should be improved in multiple aspects and the defects should be prevented. To improve the quality of the project and reduce the probability of the occurrence of inherent defects, the construction market can be scientifically managed and effectively regulated by the relevant departments. In addition, insurance companies can also ensure the effective response and repair of defects after they occur through reasonable insurance coverage for inherent defects in engineering quality.

### 4.3 Construction quality inherent defects failure probability insurance application

In this paper, by comparing other more mature insurance policies and their development history, it was found that to better cope with the risk of insurance against inherent defects, insurance companies are required to examine the probability of failure of inherent defects and then further determine insurance elements such as premium and initial reserve. The probability of failure of construction quality defects can effectively reflect the possibility of insurance claims arising from a project and provide model tools for insurance actuarial analysis.

Because the failure probability of inherent defects in construction quality was calculated in the preceding section of this paper, the failure probability of different construction project can be simulated and analyzed based on this failure probability. The binomial distribution describes the number of successes in m Bernoulli experiments [53], and because the probability of the Bernoulli test is unique, the binomial distribution can be used to fit inherent defects in construction quality and insurance claims. Assume that the claims of insurance for inherent defects of engineering quality obey binomial distribution B(m,p), where m is the number of policies covered and p is the failure probability of inherent defects of construction quality, so there are p=0.1492. By the nature of the binomial distribution, the expectation of claims for IDI is E(N)=mp, and the variance is Var(N)=mp(1−p). When the parameter m is large enough, the binomial distribution can be approximated as obeying the Poisson distribution, and the parameter of the Poisson distribution λ=mp [55], With the continuous development of engineering quality inherent defects insurance, the Poisson distribution can be used to approximate the binomial distribution, so the claim frequency parameter of engineering quality inherent defects insurance is determined λ=0.1492m. In order to better carry out the empirical analysis of IDI application, this paper obtains the claims data of People Insurance Corporation of China (hereinafter referred to PICC) through field research. The claim data are summarized under the condition of PICC informed and consent. The insurance claims data of PICC in Shanghai in 2021are shown in Table 8:

**Table 8:** Claims details of each engineering division and subdivision in Shanghai in 2021.

Scope of claims	Claims number	Proportion	Claim amount (yuan)	Proportion	Average claim amount (yuan)
10-Year Term Foundation and Main Structure	10	0.80%	134444.00	1.25%	13444.40
5-Year Term Waterproofing and Insulation	817	65.73%	7219099.04	67.30%	8836.11
5-Year Term Window and Door	1	0.08%	6150.00	0.06%	6150.00
5-Year Term Wall and Ceiling Plastering Layer	2	0.16%	8366.00	0.08%	4183.00
2-Year Term Electrical, Water Supply and Drainage	145	11.67%	1842486.87	17.18%	12706.81
2-Year Term Window and Door	83	6.68%	241773.00	2.25%	2912.93
2-Year Term Wall and Ceiling Plastering Layer	44	3.54%	282834.99	2.64%	6428.07
2-Year Term Renovation	141	11.34%	990910.30	9.24%	7027.73
Total	1243	100.00%	10726064.20	100.00	8629.17

It can be seen from the claim data in Table 8 that the sub-items with the highest frequency of problems are waterproofing and insulation, which account for about two-thirds of the prime minister's claim settlement. In the practice of IDI, it is necessary to implement detailed risk prevention and control to ensure construction projects' thermal insulation and waterproofing elements. In addition, sub-projects such as electrical, water supply, drainage and renovation also have a high frequency of occurrence. It should also be identified and controlled as the main risk point of IDI. It is worth noting that although the foundation and main structure has a higher average claim amount, the risk to IDI is not obvious due to the frequency of occurrence is too low [56].

From the perspective of insurance actuarial practice, the amount of insurance claims, that is, the degree of damage, can be considered to obey the exponential distribution [55], and its probability density function is $f\left(x\right)=\frac{1}{\theta }e^{-\frac{x}{\theta }}$ ,According to the nature of the exponential distribution, its expectation is the inverse of the parameter [53], so the parameter can be determined by borrowing the average payout per policy of insurance. From insurance companies’ insurance statistics, the insurance payout parameter for inherent defects in engineering quality is θ≈8629.17。

Because the number of claims of IDI approximately obeys the Poisson distribution, the entire claim process can be regarded as obeying the compound Poisson distribution. The insurance premium setting is often determined by multiplying the expectation of its claims by the safety factor, as shown in equation (32):

(32) it=(1+σ)E[S(t)]

And the claim process S(t) obeys the compound Poisson distribution, according to the nature of the compound Poisson distribution, the expectation of the compound Poisson distribution is equal to the product of its own two distributions, that is, E(S)=E(X)⋅E(N) [55], so the expectation of the engineering quality inherent defects insurance claim process is E[S(t)]=λ⋅θ≈1827.47m. Thus, the premium setting for engineering quality inherent defects insurance can be calculated as equation (33):

(33) it=(1+σ)E[S(t)]=1827.47m(1+σ)

According to the bankruptcy theory of insurance, there is $P\left(t\right)=u_{0}+it-S\left(t\right)$ [57] is introduced into the insurance premium calculation formula and equation (34) can be obtained:

(34) $$P\left(t\right)=u_{0}+it-S\left(t\right)=u_{0}+1827.47m\left(1+\sigma \right)-S\left(t\right)$$

Based on the idea of sample normalization, a new process function $Z=\frac{S-E\left(S\right)}{\sqrt{Var\left(S\right)}}=\frac{S-\lambda E\left(X\right)}{\sqrt{\lambda E\left(X^{2}\right)}}$ can be constructed and an approximate derivation can be made by using the uniqueness of the moment mother function of the probability distribution. Through the evolution of the moment mother function as well as the expansion, it can be concluded that the new process function approximately obeys the standard normal distribution [53] when the parameter m is sufficiently large. Thus, the insurance risk case can be determined by the standard normal distribution table, and the insurance risk cases are as shown in equation (35):

(35) $$p\{S\left(t\right)\geq it+u_{0}\}=p\{S\left(t\right)\geq 1827.47m\left(1+\sigma \right)+u_{0}\}=p\{\frac{S\left(t\right)-E\left[S\left(t\right)\right]}{\sqrt{Var\left[S\left(t\right)\right]}}\geq \frac{u_{0}+1827.47m\sigma }{\sqrt{2\lambda \theta^{2}}}\}=1-\Phi \left(\frac{u_{0}+1827.47m\sigma }{\sqrt{2\lambda \theta^{2}}}\right)=1-\Phi \left(\frac{u_{0}+1827.47\sigma \sqrt{m}}{4613.18}\right)$$

**Fig. 12:**
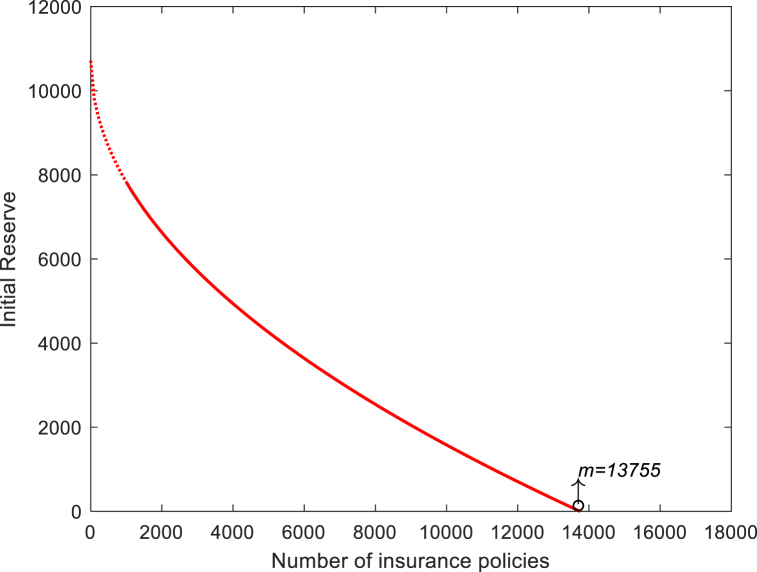
Initial insurance reserve as a function of the number of insurance policies.

According to the requirements of insurance practice, σ is taken as 0.05, and the initial insurance reserve required under a fixed number of policies can be solved inversely when the insurance risk is controlled at the 1% level, such that $p\{S\left(t\right)\geq it+u_{0}\}=0.01$. Then, the relationship between the number of policies and the minimum required initial insurance reserve is shown in Fig. 12:

The solid red line is the minimum initial reserve required to satisfy the insurance risk of less than 0.01 for that number of insurance policies, and the upper right part of the curve is the insurance feasibility domain for which the risk is satisfied. In the process of model construction, many derivations and evolutions are based on a large sample; thus, the number of insurance policies m≤1000 does not conform to the distribution of the function when the number of insurance policies is plotted with a dashed line to ensure the completeness of the function and cannot be analyzed according to the characteristics of the function in the dashed part. The graph of the initial insurance reserve as a function of the number of insurance policies shows that as the number of insurance policies increases, the average initial reserve required per insurance policy gradually decreases and the risk gradually stabilizes. At the insurance risk level of 0.01, the required insurance initial reserve is 0 when the number of insurance policies m exceeds 13755, at which time insurance can be guaranteed to keep the insurance risk below 0.01, without the initial reserve.

## 5 Conclusions

Through the bibliometric analysis of the influencing factors of inherent defects, it can be seen that the influencing factors of inherent defects can be roughly summarized into three categories: member factors, natural environment factors and policy environment factors. On the basis of determining the performance function of inherent defects fitted by PSO-LSSVR model, the reliability index and failure probability of inherent defects are derived in this paper, by combining the method of structural reliability index in engineering. The reliability index of the inherent defects is 1.039 and the failure probability is 0.1492, which can provide theoretical support for the pricing and standard setting of insurance companies. By defining the failure probability of inherent defects, this paper uses binomial distribution and exponential distribution to fit the number of claims and the distribution of claims for inherent defects insurance respectively. And then the risk model of engineering insurance is constructed by the ruin theory of insurance. It can be found that with the increase of the number of insurance policies, the initial reserve required for each insurance gradually decreases, and the risk gradually tends to be stable. According to the insurance data of Shanghai, when the policy number m exceeds 13755, the required initial insurance reserve is 0, which can fully meet the insurance risk requirements.

This paper regards the influencing factors as function variables, and constructs the index evaluation system of inherent defects. Nearly 100 pre-evaluation data of third-party quality inspection agencies in 12 cities in China were collected, and the performance function of inherent defects was fitted by PSO-LSSVR model. And then, the performance function of the inherent defects is determined, and the validity of the model fitting is proved according to the comparative analysis of the general multiple regression model. This model has great value in the management and decision-making of IDI, and can also be applied to all kinds of types of construction projects duo to the paper does not involve the characteristics of different types of construction projects.

In order to better manage the risk of IDI, the following measures should be taken. First of all, the underwriting loss mechanism of insurance should be improved. Clarifying the specific scope of insurance underwriting and establishing a more complete claims system to reduce the overall degree of claims can reduce the risk of insurance operation from the level of claims. What’ more, the insurance actuarial system and related databases should be improved. It is best to develop a more intelligent determination of IDI insurance rates from the perspective of big data. At the same time, it comprehensively sorts out the possible impact of relevant influencing factors on insurance premium rates, constructs a more comprehensive and accurate IDI ratemaking model, and reduces the possibility of insurance bankruptcy from the level of premium income. Finally, the establishment of insurance risk prevention system, including the initial reserve. It is necessary to set an appropriate initial insurance reserve so as to effectively improve the ability of IDI to resist risks while ensuring the profits of insurance companies and comprehensively reduce IDI risks.

However, there are still many directions for improvement in this paper. The index evaluation system constructed in this paper can be further improved, and more effective data can be obtained. Further, the derivation of the risk model depends more on the existing probability distribution, and the fitting of the risk process of the IDI project needs to be improved. It can be considered to use a more accurate distribution to fit the IDI project process in the next step of research.

## Data availability

The data used to support the findings of this paper are included within the article.

## CRediT authorship contribution statement

**Zeyu Chen:** Writing – original draft, Visualization, Validation, Software, Methodology, Funding acquisition, Formal analysis, Conceptualization. **Xikang Yan:** Writing – review & editing, Validation, Supervision, Resources, Project administration, Methodology, Funding acquisition, Conceptualization. **Lida Wang:** Writing – review & editing, Visualization, Validation, Investigation. **Qinyu Luo:** Writing – original draft, Data curation. **Yunhan Yan:** Investigation. **Tian Qiu:** Writing – original draft. **Peng Cheng:** Software.

## Declaration of competing interest

The authors declare the following financial interests/personal relationships which may be considered as potential competing interests:Zeyu Chen reports financial support was provided by Hebei Provincial Department of Housing and Urban-Rural Development. If there are other authors, they declare that they have no known competing financial interests or personal relationships that could have appeared to influence the work reported in this paper.
